# AWOSE - A Process Model for Incorporating Ethical Analyses in Agile Systems Engineering

**DOI:** 10.1007/s11948-019-00133-z

**Published:** 2019-10-07

**Authors:** Benjamin Strenge, Thomas Schack

**Affiliations:** Cluster of Excellence ‘Cognitive Interaction Technology’ (CITEC), Neurocognition and Action Research Group, Faculty of Psychology and Sports Science, Bielefeld University, Inspiration 1, 33619 Bielefeld, Germany

**Keywords:** Human factors, Ethics, MEESTAR, Worth, Agile development

## Abstract

Ethical, legal and social implications are widely regarded as important considerations with respect to technological developments. Agile Worth-Oriented Systems Engineering (AWOSE) is an innovative approach to incorporating ethically relevant criteria during agile development processes through a flexibly applicable methodology. First, a predefined model for the ethical evaluation of socio-technical systems is used to assess ethical issues according to different dimensions. The second part of AWOSE ensures that ethical issues are not only identified, but also systematically considered during the design of systems based on information and communication technology. For this purpose, the findings from the first step are integrated with approaches from worth-centered development into a process model that, unlike previous approaches to ethical system development, is thoroughly compatible with agile methodologies like Scrum or Extreme Programming. Artifacts of worth-centered development called Worth Maps have been improved to guide the prioritization of development tasks as well as choices among design alternatives with respect to ethical implications. Furthermore, the improved Worth Maps facilitate the identification of suitable criteria for system evaluations in association to ethical concerns and desired positive outcomes of system usage. The potential of the AWOSE methodology has been demonstrated in the context of a technical system (smart glasses for cognitive assistance) that supports elderly and people with particular handicaps.

## Introduction

The discovery of nuclear fission by German scientists Otto Hahn and Fritz Strassmann during World War II led to research on nuclear chain reactions culminating in the creation of the first nuclear weapons by the U.S. during the Manhattan Project. A few decades later, after school shootings around the millennium change, authorities were quick to allege that computer games like Counter-Strike had a negative psychological impact on the killers, despite a glaring lack of scientific evidence supporting these claims. More recently, the widespread use of (web-based) social networks not only raised privacy concerns, but also created unwanted phenomena like “cyberbullying” or “cyberharassment”. All of these chains of events make it abundantly clear that scientists and engineers are well advised to assess long-term consequences of their research and development projects carefully. In most cases, even the direst worst-case impacts on society may not match the potential of weapons of mass destruction. Nonetheless, the current consensus among researchers is that ethical, legal and social implications (ELSI) are an important aspect of all science and engineering endeavors. A substantial body of current research is concerned with finding proper ways of educating and sensitizing engineers to ethics (e.g. Gelfand 2016; Miñano et al. 2017; Murphy and Gardoni 2017; VanDeGrift et al. 2017; Bairaktarova and Woodcock 2017; Cheruvalath 2017). However, less extensive guidance has been offered regarding approaches to systematic handling of ethical issues during actual development processes for systems based on information and communication technology (ICT). As of yet few specific guidelines are offered on how to assess and handle ethical issues during the day-to-day work in agile development processes that are characterized by transient requirement definitions and limited overall predictability. This constitutes a pressing issue since an absence of explicit ethical considerations may lead to suboptimal adoption of new technologies such as intelligent assistive systems (Ienca et al. 2017). There are two main issues to solve: (1) How to identify and assess ethical implications, and (2) how to handle these during system development.

Arguably the most well-known approach that aims at tackling these issues is the Value-Sensitive Design (VSD) methodology (Friedman et al. 2008), which has been applied in more or less structured ways in many projects (e.g. Friedman et al. 2008; van den Hoven et al. 2015; Royakkers and Steen 2017; Umbrello and De Bellis 2018). VSD is presented as a tripartite methodology comprising three types of value-related “investigations” (conceptual, empirical, and technical), which “overlap and intertwine so that boundaries between them are blurred” (Davis and Nathan 2015, p. 32). Publications on VSD (e.g. Friedman et al. 2008) claim that stakeholders and benefits/harms for these must be identified, mapped onto corresponding values, and should be explicitly related to relevant design trade-offs. Recently (see also Manders-Huits 2011; Yetim 2011; Reijers et al. 2017) a suitably comprehensive overview was published about which methods and tools could be applied to these ends (Friedman et al. 2017). However, the selection and systematic integration of appropriate methods in agile development processes remains an underspecified aspect in the “official” VSD literature by Batya Friedman and colleagues. An elaborate, well-defined methodology that connects VSD approaches with IT system design processes has been proposed by Spiekermann (2015). While her “ethical system design lifecycle” (E-SDLC) fits classical, plan-driven development processes particularly well, Spiekermann (2015, p. 164) claimed “agile software development can [also] be used in ethical system design. The only thing that needs to be fulfilled is that earlier system design phases get the requirements and architecture right up front.” This may not be feasible in many projects, since development teams often choose agile approaches when they expect frequent requirements changes and commonly re-factor the code to adjust the architecture correspondingly (see also Beck 2000).

Conversely, a large number of well-defined process models have been developed for combining agile development approaches like Extreme Programming (Beck 2000) or Scrum (Schwaber 1997) with user-centred design methods (e.g. Holzinger et al. 2005; Memmel et al. 2007; Singh 2008; Obendorf and Finck 2008; Lee et al. 2009). These user-centred design methodologies strongly emphasize the importance of usability as a product characteristic, but disregard any ethical aspects that do not happen to coincide with specific user requirements regarding effective, efficient and satisfying system usage.

This article proposes a structured approach to filling these gaps concerning agile development processes by incorporating ethically relevant criteria through a flexibly applicable methodology called Agile Worth-Oriented Systems Engineering (AWOSE). The AWOSE methodology is based on preliminary work from a computer science master’s thesis (Strenge 2013) and has been extended and refined during the research project “ADAMAAS – Adaptive and Mobile Action Assistance” (Essig et al. 2016). The goal of project ADAMAAS was to use smart glasses and related augmented reality setups in combination with psychological measures to provide cognitive assistance to people in daily living activities, education or work tasks. Since the project’s primary target groups comprised people who are particularly vulnerable, such as handicapped or elderly people, a careful and systematic consideration of ethical issues was particularly important. Throughout this article, the core concepts of AWOSE shall be illustrated by examples from its application in the ADAMAAS project.

In AWOSE’s first part, a multi-dimensional model for the ethical evaluation of socio-technical arrangements (MEESTAR, Manzeschke et al. 2015) is used to identify and assess ethical issues on an individual, organizational and social level, as well as according to a standardized set of dimensions, such as privacy, participation or safety. Each potential issue’s severity is evaluated according to a four-level scale that ranges from “*completely harmless*” to “*should be opposed from an ethical viewpoint*”. As a result, detailed information about relevant ethical issues regarding the socio-technical system is gained. The second part of AWOSE ensures that these ethical issues are not only identified, but also adequately considered during system development process by integrating the MEESTAR-based analyses with approaches from worth-centered development. Special artifacts from the worth-centered development methodology called Worth Maps have been extended and improved to combine project management tools and engineering methods, which guide the regular prioritization of development tasks as well as systematic choices among design alternatives with respect to ethical implications. Furthermore, the improved Worth Maps facilitate the identification of suitable criteria for system evaluations in association with ethical aspects and explicitly relate these to desired user experiences and positive outcomes of system usage. Finally, this article presents a process model for structuring and organizing both parts of the AWOSE methodology, which combines user experience, engineering, and ethical assessments, and is compatible with well-known agile methodologies like Scrum or Extreme Programming.

## Identification and Assessment of Ethical Issues

The first part of AWOSE can start whenever a suitably representative description of the technology, technical system, or product has been developed. (In the following, this article will refer to a “system” being developed, and the respective “system vision”, but the statements generally hold for technologies and product developments as well.) The description may have any form, e.g. a simple textual description, graphical sketches, diagrams, mockups, or a usable prototype. The degree of how detailed the description should be poses a trade-off, as is the case for many other human-centered system design methods: The less detailed the description, the less reliable will any assessment be that is based on it. The more detailed the description, the fewer degrees of freedom may remain to adjust the design. In general, it is advisable to start with an early version and continually re-iterate the assessment process as deeper knowledge regarding aspects and features of the system and its environment, including users and other stakeholders, becomes available.

**Fig. 1:**
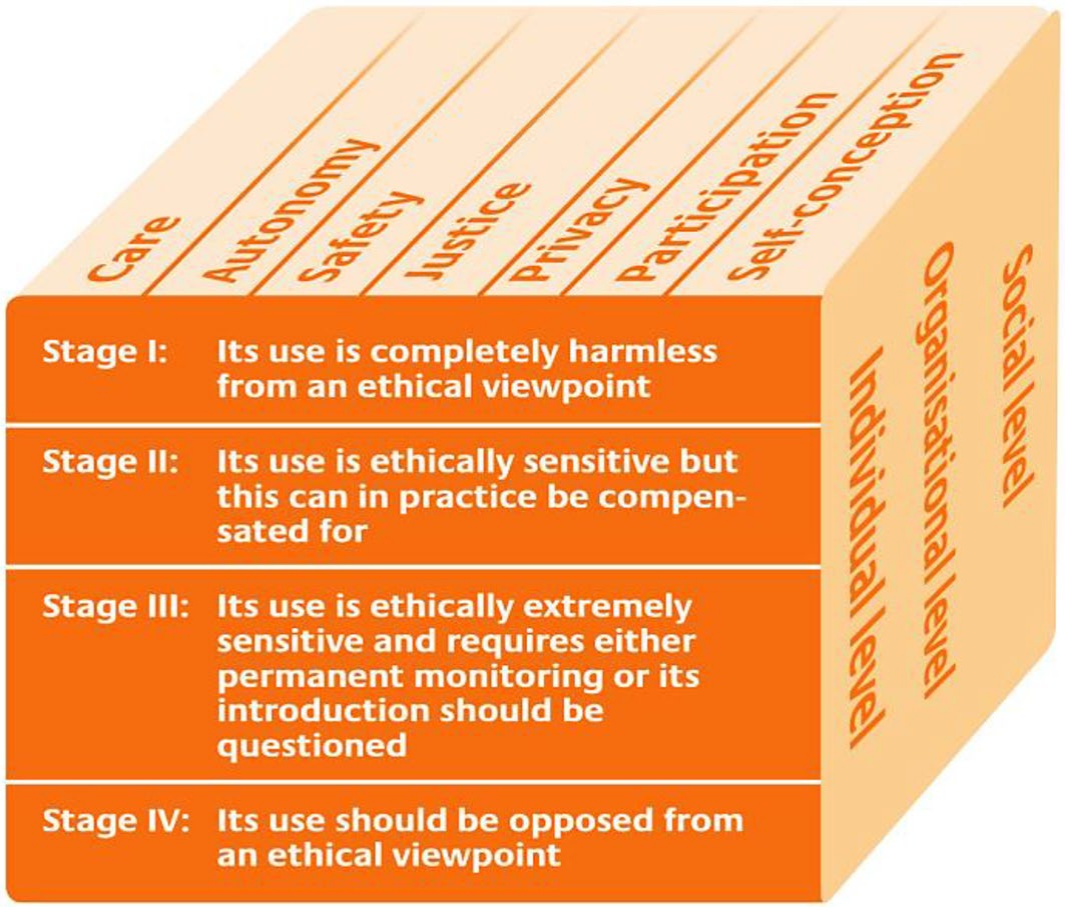
The multi-dimensional model for the ethical evaluation of socio-technical arrangements (MEESTAR) from Manzeschke et al. (2015) is used as the first part of AWOSE. (Color figure online)

In order to identify ethical issues, MEESTAR, a “multi-dimensional model for the ethical evaluation of socio-technical arrangements” created by German philosopher, theologian and anthropology professor Manzeschke et al. (2015), is used. MEESTAR was originally developed for analyses of “age-appropriate assisting systems”, which comprise a broad range of (socio-)technical systems that are supposed to be used primarily by elderly people to help them live autonomously in their own homes. However, it has also been applied in contexts such as assistance for young people with disabilities, telemedicine, and systems for working environments (Manzeschke 2015). MEESTAR provides a reference framework to structure discussions about system-related ethical aspects, ideally in the form of interdisciplinary workshops, with respect to a set of pre-defined dimensions (Fig. 1). As a preparatory step, the model and the system description, including the intended context of use, are presented to all workshop participants. MEESTAR then requires systematic consideration of ethical issues related to seven dimensions (care, autonomy, safety, justice, privacy, participation and self-conception) on an individual, organizational, and social level.

The seven ethical dimensions have been derived from theoretical ethical work as well as a series of qualitative interviews (Manzeschke et al. 2015). These dimensions are not meant to serve as guidelines from which ethical judgments could be derived, but to help evaluators “to identify and allocate one or more ethical issues in an actual scenario” (Manzeschke et al. 2015, p. 14). Extensive definitions and corresponding examples for all seven dimensions have been provided by Manzeschke et al. (2015). Nevertheless, there is certainly some degree of overlap and fuzziness concerning the mapping of identified ethical issues to these dimensions. This does not seem to compromise the usefulness or applicability of the model, but rather fosters prolific discussions and reflection of each issue. Actually, supporting brainstorming and discussion is the main purpose of referring to MEESTAR’s dimensions, whereas structuring the resulting output may be considered a secondary benefit. Generally, it makes little sense to understand any ethical “dimensions” or “values” as objective, absolute and stable constructs for a variety of reasons. Due to the associative nature of human cognition, everyone will understand a term like “privacy” or “justice” in a slightly different way (see also Umbrello 2018a), and morality changes over time and interacts with technology (Boenink et al. 2010). It is therefore highly important that a sufficiently large and (cognitively) diverse set of people participate in the MEESTAR workshops. In a similar vein, MEESTAR’s co-creator Weber (2018) acknowledged that the proposed dimensions, combined with preconceptions regarding their meaning, might influence workshop participants and their judgment. He suggested that VSD methods and literature could be used to “systematically identify moral dimensions for MEESTAR” related to a specific project context (Weber 2018, p. 260). However, he also noted that many projects might not have enough resources to do so. Furthermore, MEESTAR’s default dimensions can be mapped to the four basic principles of biomedical ethics (i.e. autonomy, beneficence, non-maleficence, and justice; Beauchamp and Childress 2006), which have been widely used in ethical evaluation methods (Weber 2018; Reijers et al. 2017). In order to support its agile orientation, AWOSE uses the abovementioned seven dimensions by default, but the methods for identifying “worth”, which are described in this article’s section about “Worth Mapping basics”, as well as other appropriate sources can be used to inform and adjust this set if necessary. This seems especially important if the developed system’s properties deviate significantly from the properties of age-appropriate assistance systems that MEESTAR’s default dimensions primarily aim to cover. Generally, MEESTAR focusses on addressing ethical concerns related to the wellbeing of human stakeholders. However, issues related to other lifeforms (plants, animals, etc.) are not at all explicitly covered. The analyses should therefore be broadened to include nature-related implications. Nowadays, perceptions and appreciation of non-human life differ widely, ranging from demanding equal treatment of all forms of life, to claims that only human requirements matter. As Steven Umbrello, Managing Director at the Institute for Ethics and Emerging Technologies, noted: “Contemporary scholarship on metahumanisms, particularly those on posthumanism, have decentered the human from its traditionally privileged position among other forms of life” (Umbrello 2018b, p. 3). A consideration of nature-related aspects tends to increase awareness of issues that are undoubtedly as important for the long-term development of human societies as they are for earth’s overall ecosystem, e.g. issues related to sustainable production, operation, and maintenance of systems.

While the AWOSE methodology requires at least one MEESTAR workshop as soon as the system vision is available, it is often advisable to schedule several iterations and update the list of ethical issues over time as more and more is known about the system and its context of use. During the ADAMAAS project’s 3-year funding period, five half-day MEESTAR workshops have been organized with an average of nine to ten participants, including representatives of the project partners and stakeholders. Since ADAMAAS could be considered as an age-appropriate assistance system, MEESTAR’s default dimensions were used. Retrospectively the initial list of relevant ethical issues had converged towards a reasonably stable set after the third workshop.

### Referring to Individual, Organizational, and Society Levels

Depending on the total number of workshop participants, the group can be split into subgroups tasked with working on the individual, organizational, or society level. From a user-centered design perspective, the individual level may be considered the most important, but Manzeschke et al. (2015, p. 20) argued that not just individuals have to be responsible for their actions, but also corporative entities such as companies, and that a social level of responsibility must be discussed as well. While the relevant organizations and societies are usually identified with relative ease, in the frame of AWOSE a meaningful reference to individuals must be established using specific stakeholder models. Arguably, Personas (Cooper 2004; Pruitt and Adlin 2006) are most suitable for this task due to a distinct set of properties:

- they constitute generalizations from real individuals such that a small set of Personas represents large groups of users and other relevant stakeholders,
- they are highly detailed and can be specifically based on relevant types of data from market or user research, and
- they effectively exploit well-developed human capabilities such that designers and developers can easily extrapolate the persona descriptions to infer likely behaviors of the represented “persons” in a given situation (Pruitt and Grudin 2003).

In order to ensure objectivity, Persona descriptions should be derived from actual data regarding relevant stakeholder properties in a systematic and traceable way, e.g. using descriptive statistics and/or approaches based on principal component analysis or factor analysis (Sinha 2003; McGinn and Kotamraju 2008; Miaskiewicz et al. 2008), i.e. a reduction of high-dimensional data spaces (e.g. from questionnaires) to a smaller set of uncorrelated linear combinations of the original properties. However, in absence of applicable data, so-called “ad-hoc Personas” (Norman 2004), i.e. fictive descriptions of hypothetical stakeholders, can still be useful to make explicit statements and reach consensus about the targeted stakeholder groups instead of nontransparent implicit assumptions. In the ADAMAAS project, survey-based data about stakeholder characteristics could be acquired for two of three application scenarios in order to derive Persona descriptions based on statistics. For the remaining scenario, ad-hoc Personas have been created based on researchers’ observations and assumptions and then handed over to the application partner’s human resources department for validation. In all of these cases, “primary” Personas represented potential user groups, while “secondary” Personas represented indirectly affected stakeholders (e.g. users’ supervisors or managers). All Persona descriptions were then printed and handed out to MEESTAR workshop participants.

### Assessment of Ethical Sensitivity

The final step of MEESTAR consists of an evaluation of each identified ethical issue on a scale with four levels, ranging from “*completely harmless*”, “*ethically sensitive*” and “*extremely sensitive*” to “*should be opposed from an ethical viewpoint*” (Manzeschke et al. 2015, p. 14). Hereby it is important to note that each of these assessments is explicitly related to (1) one of the seven ethical aspects, (2) a specified individual, organization, or society, and (3) a specific timeframe. In AWOSE, the latter is by default implicitly defined as a snapshot of the current reality at the instant when the assessment takes place, but it may be worthwhile to consider the expected impact of foreseeable developments, especially for upcoming technologies and products with a prolonged lifespan. Since MEESTAR-related analyses in AWOSE are supposed to be conducted by an interdisciplinary group (e.g. researchers, engineers, potential users, practitioners and domain experts with different backgrounds), in many cases the initial judgments regarding each issue’s ethical sensitivity may vary. The proper way of resolving these situations obviously poses an ethical question in itself, which the original publications on MEESTAR did not cover. The pragmatic solution in AWOSE is to try first to reach a consensus on the sensitivity through discussion. If this fails, the highest severity rating chosen by any member of the interdisciplinary group is selected, i.e. the goal is to err on the side of caution.

Since its creation in 2013, MEESTAR has proven a useful instrument for identifying and assessing a broad range of ethical issues in different research and development projects. However, it does not indicate how these issues should be handled with respect to the concrete design and implementation of system components. Therefore, up to this point it remains largely unclear to engineers and developers what exactly they should do, or not do, or how they should do it, during their day-to-day work creating the system. Another limitation of MEESTAR is the sole consideration of potentially negative aspects, because it is meant to safeguard against harm as “the minimum ethical requirement” (Manzeschke et al. 2015). Whenever the potential negative consequences of a system are within a tolerable range, they must be traded off against expected positive outcomes. The second part of AWOSE aims at tackling both of these shortcomings.

## Integration with Approaches from Worth-Centred Development

The classical user-centred design and usability engineering methodologies focus on properties of users and their interaction with a system, while user experience is mainly concerned with users’ aesthetic and emotional perceptions before, during and after system usage (ISO 9241-210). Worth-centred development (WCD) (Cockton 2006) goes beyond these considerations and demands that the worth that is generated for people or organizations by (using or applying) a system should be targeted as the prime focus during development. Hereby “worth” may refer to any kind of individual or collective ethical, practical, financial, emotional, or other benefits and positive outcomes of system usage. The notion of worth even includes “unfelt needs” (Cockton 2006), i.e. worth that is not yet consciously known to or explicable by potential stakeholders. This facilitates the creation of highly innovative products whose worth may only become evident by the time they are used. American psychologist Frederick Herzberg (1964) considered human motivation as an interaction of two main factors: Motivators, whose presence generates satisfaction (e.g. appreciation or professional success), and hygiene factors, whose absence creates dissatisfaction (e.g. payment or safety). With respect to this model, WCD asks system designers to focus “*on the* worthwhile*, that is, things that will be valued, as manifested in people’s motivation, individually or collectively, to invest one or more of time, money, energy and commitment. […] In short, worth is a motivator [and] designing worth means designing things that will* motivate *people to buy, learn, use or recommend an interactive product, and ideally most or all of these.*” (Cockton 2008a, p. 168) In this sense, the focus of WCD seems to be contrary to that of MEESTAR at first glance. WCD’s creator acknowledged that weighting of positive and negative aspects is required, such that the resulting system design “delivers sufficient value to its intended beneficiaries to outweigh costs of ownership and usage” (Cockton 2008a, p. 60). Contrary to the precursory framework called “value-centred design” (Cockton 2005), the existing publications on WCD do not include a well-defined process model, but rather constitute a set of approaches to apply throughout development. Arguably, the most important and widely used of those approaches is the Worth Map, a specific type of diagram that supports and structures systems design with respect to WCD’s premises.

### Worth Mapping Basics

In market research, means-end chains describe the (expected) causal connections between product features, customers’ emotions and their motivation for buying. WCD adapts this approach to the principle of “designing as connecting”: Dependencies and connections between different system designs, usage, user experiences, stakeholders, and evaluation metrics are analyzed and expressed by connecting the respective elements (Cockton 2009a, b; Cockton et al. 2009a, b), e.g. visually in a diagram consisting of boxes and arrows. In WCD and AWOSE, the elements of means-end chains can be materials and other components, features, qualities, and, finally, worth of a specific system. Different methods can be used to identify the elements of means-end chains with respect to a specific system:

#### **Brainstorming**

about human needs, desires, aversions, motivations, habits and technical possibilities as well as experiences with comparable systems and current trends can be conducted by an interdisciplinary team (Cockton 2008b).

#### **Laddering**

is a technique originating in clinical psychology where it is used as an instrument to find out about the understanding that people have regarding their social relationships by asking them to describe people meaningful to their own life and then recursively querying about the meaning of constructs used in their description (Cockton 2008a). This yields extensive information about people’s personalities and values. In marketing, laddering is used to uncover the relation between personal values and the perceived benefit of products. To this end, customers are asked to name product attributes that are important to them. Afterwards they are recursively asked why these attributes are important, “*repeating this ascent up the ladder until a consumer can only say that something really matters to them*” (Cockton 2008c, p. 293). The same principle can be applied in WCD to identify means-end chain elements and their association related to a system.

#### **Sentence Completion**

tests are semi-structured, projective surveys that have been applied e.g. as personality tests (Holaday et al. 2000), for determining managers’ motivation (Brief et al. 1977), and in consumer research (Donoghue 2000). Participants are asked to finish given incomplete sentences according to their own first association. Such an incomplete sentence could be: “*Professionally, the most important thing for me is…*” It has been reported that sentence completion using incomplete sentences derived from a set of general and project-specific human values yielded better results than interviews and, despite the predetermined beginning of the sentences, is open enough not to subject participants to priming effects (Cockton et al. 2009b).

While brainstorming has the potential to generate any kind of means-end chain elements and laddering identifies complete means-end chains starting from system features upwards, sentence completion aims at uncovering people’s most important values and motivators, i.e. system design goals in the form of intended worth. In AWOSE, any of the abovementioned methods, or a combination thereof, can be used to identify worth in the sense of motivators and desired positive outcomes of system usage. In the ADAMAAS research project, the intended worth was originally derived from the predefined project goals and extended through brainstorming in interdisciplinary groups and stakeholders surveys.

All identified means-end chains and possibly unconnected elements are then combined into a single diagram, the Worth Map of a system. Worth Maps are the core artefacts of WCD. They serve as a basis for discussion, to represent development goals and means for accomplishing them, and for planning evaluations. In order to create a Worth Map, the means-end chains are merged at common elements (if such exist) and complemented with isolated chains and elements. In AWOSE, the initial Worth Maps are iteratively refined and extended during development. Depending on the scope and complexity of the system, Worth Maps can become quite large and complex as well. It is therefore advisable to use software tools that facilitate making changes and adjustments to the diagrams with levity. Microsoft Office Visio has been recommended for this purpose (Cockton et al. 2009b), with LibreOffice Draw constituting a serviceable open source alternative, and a dedicated tool for Worth Mapping was developed in a computer science diploma thesis at Paderborn University (Strotmeier 2001).

### Integrating Ethical Issues in Worth Maps

**Fig. 2:**
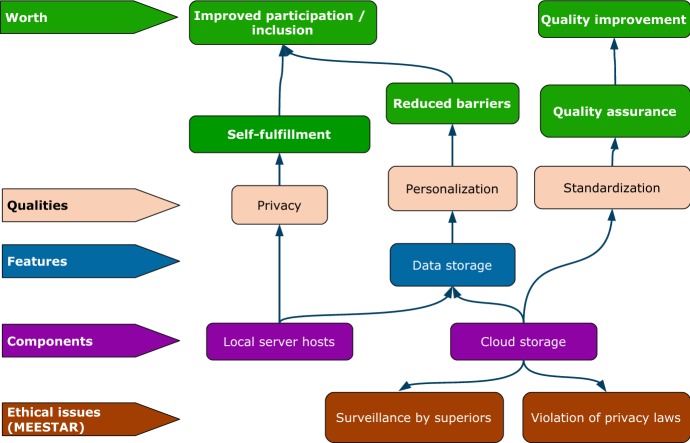
Excerpt of a Worth Map sketch from ADAMAAS, a research project on an adaptive assistance system, representing a design decision regarding storage of data about users for the purpose of individualized adaptation. Purple boxes describe system components, blue boxes list possible features, orange boxes show qualities, and green boxes indicate worth, i.e. positive outcomes of the system. Red boxes in the bottom row represent ethical issues identified with MEESTAR. (Color figure online)

It was explicitly not the original aim of WCD to avoid negative outcomes of a system’s usage at all costs (Cockton 2012), but rather it was to focus on worthwhile outcomes. Arguably, it depends on the assessed severity of ethical issues in how far they should prevent or restrict the usage of a system or its components. Additionally, when ethical issues apply only to some specific design variants or ways of implementing a given feature, often less critical alternatives can be chosen. This is highly important information for designers and engineers to keep in mind during development. The structure of Worth Maps is well suited to facilitate this. To this end, the Worth Maps in AWOSE are extended by integrating the output from the first part based on MEESTAR, i.e. an additional layer of elements is added to the bottom of the Worth Map diagram, representing all ethical issues that have been identified (see bottom row of Fig. 2). The workshop participants from the first part then come together again to investigate the associations between the Worth Map elements (e.g. system components and features) and ethical issues. These connections are graphically indicated with arrows in the Worth Map.

### Increasing the Expressivity of Worth Maps by UML Integration

There are two basic types of connections between Worth Map elements, which indicate that positive or negative outcomes are either enabled (solid lines) or disabled (dashed lines), as described by British design theory professor Gilbert Cockton et al. (2009b). These simple types of connections are often insufficient for describing the interrelations of complex systems. Therefore, Worth Map diagrams in AWOSE use relationship notations from Unified Modeling Language (UML) structure diagrams when required, i.e. specific types of lines and arrows to indicate relations between elements such as *implementation*, *dependency*, or *composition*. This is especially useful for the layers containing technical descriptions (e.g. system components and features). With proper tool support, a broad range of UML diagrams can directly be integrated as elements or layers into Worth Maps. Zooming in and out of such extended Worth Maps as a project’s master diagram can help increase designers’ and developers’ awareness of the “bigger picture”, i.e. each system component’s relation to features, qualities and, finally, desired positive outcomes of system usage, as well as ethical issues that should be considered.

### Ethical and Worth-Related System Evaluation

Designing and developing systems in a way that fulfills specified goals is a basic concept of engineering (Butler 1985). Process models like the usability engineering lifecycle (Mayhew 1999) and the human-centred design process from ISO 9241-210 require the definition of specific goals regarding usability and corresponding requirements as the basis for system evaluations. This highlights the importance of usability and user experience as non-functional requirements within these frameworks. The definition of specific usability goals facilitates the planning of usability evaluations (Quesenbery 2001) and may even guide the overall system design process by establishing the most important values and goals the resulting product shall fulfill (Quesenbery 2003). In iterative design processes, usability metrics can be used to decide if further iterations are necessary (Whitefield et al. 1991). Leading IT companies like IBM, Microsoft, and Google use the evaluation results as a formal basis for deciding upon product release (Beauregard and Corriveau 2007). The consensus is that the definition of specific goals and corresponding evaluation is beneficial. However, different approaches have been taken in deciding how to define suitable evaluation criteria. In the practice of usability engineering, evaluators often resort to generic measures that are easily operationalized, e.g. the time users require for task completion, or the number of errors they make when interacting with the system, independent of the actual relevance of these measures in a given context. Instead, project-specific proprietary measures should be defined with respect to each system, its context of use, and the overall goals (Cockton 2008c; Beauregard and Corriveau 2007).

A central premise of WCD and AWOSE is that usability does not carry inherent worth, but rather that it is often a necessary means to superordinate ends. In AWOSE, the definition of metrics for system evaluations works as follows: On the one hand, the Worth Map elements are analyzed starting at the top level (worth) and then possibly going down to lower levels of system qualities, features, or components, if and only if the associated higher-level elements cannot be measured. As an example, imagine an assistance system that is supposed to help people with handicaps to learn how to perform working steps more quickly and independently from their teachers. The faster learning rate and independence may be considered positive outcomes or worth. Therefore, if it was possible to measure the users’ degree of success in learning the working task and their independence before and after introducing the assistance system, the generated worth could be assessed without doing classic usability tests with the system. However, it may be that participants cannot be exposed to a system prototype of unknown quality, because it might confuse and irritate them, or a proper assessment of their success at learning new working tasks would require too much time and resources. In this case, evaluation may need to resort to lower-level metrics like the understandability of the wording and quality of icon design of the assistance system’s user interface.

On the other hand, AWOSE also requires system evaluators to consider potential ethical concerns. Most of these, and coercively those with critical severity ratings, should not make their way into system implementations in the first place. For the remaining issues, evaluation metrics must be defined. For example, imagine again the abovementioned assistance system. An ethical issue might be that users succeed well in performing the working tasks when using the system, but rely heavily on its assistance instead of learning the task on their own. This would indicate that an unwanted dependence on technology has been induced, which would be contrary to the goal of facilitating learning processes. A corresponding evaluation metric for this issue could be to regularly assess and compare users’ task performance with and without the assistance system.

Now that the ingredients and rationale of AWOSE have been outlined, the next section proposes a process model to structure agile development endeavors with respect to ethical and worth-related aspects.

## An Agile Process Model

While traditional software engineering process models like the waterfall model are divided into discrete, sequential phases (Royce 1987), proponents of agile methodologies like Scrum’s inventor Ken Schwaber have rejected this: “*The stated, accepted philosophy for systems development is that the development process is a well understood approach that can be planned, estimated, and successfully completed. This has proven incorrect in practice*.” (Schwaber 1997, p. 1) In order to cope with uncertainty and limited plannability, and react flexibly to changing requirements, agile approaches do not consider system features, architectures and components as static and fixed throughout development. Instead, planning of development tasks is limited to short timeframes and continually readjusted (Beck 2000). Since results of user tests and correspondingly required design changes are hardly predictable beforehand, agile development methodologies generally accord very well with the requirements of human-centred design processes (Holzinger et al. 2005; Memmel et al. 2007; Singh 2008; Obendorf and Finck 2008; Lee et al. 2009). While the previous proposals for integrating human-centred aspects and agile methods mainly focused on conventional usability and user experience, AWOSE establishes worth-related and ethical aspects as the primary concern.

**Fig. 3:**
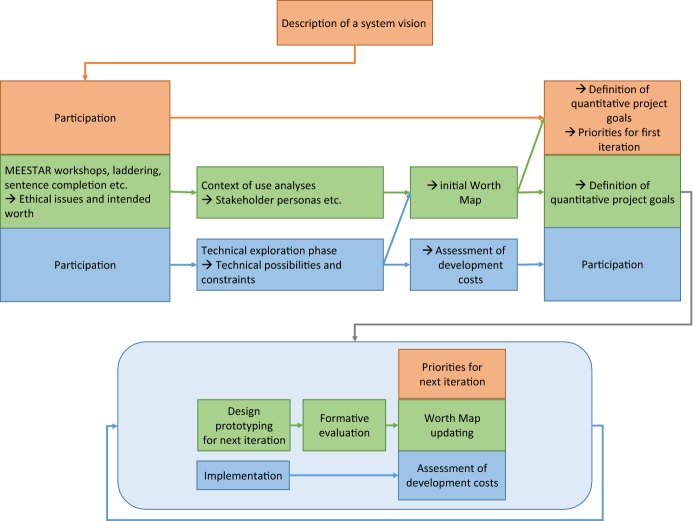
The AWOSE process model. Orange boxes contain responsibilities of the customer or product owner, green boxes show responsibilities of the worth designer, and blue boxes indicate responsibilities of developers. (Color figure online)

The AWOSE process model (Fig. 3) assigns responsibilities to three different roles:

- The *customer* plays a similar role as in the Extreme Programming (XP) (Beck 2000) and can be related to Scrum’s concept of a “product owner”. He or she should be available for the system development team during the whole project. However, the role can be assumed by different individuals over time, with the additional benefit of having different people as test users for “quick-and-dirty” formative evaluations.
- The *worth designer* fulfills comparable duties as usability engineers, interaction designers, or user experience specialists, but aims to support the worth- and ethics-oriented goals.
- The *developer* is responsible for technical planning and system implementation.

Note that in large projects usually a handful of people will share the roles of worth designers and developers. The (non-iterative) preproduction phase of AWOSE starts with the definition of a “system vision” by the customer. As discussed in the context of AWOSE’s first part, the granularity of the resulting description can vary and depends on project properties. The worth designer then organizes an interdisciplinary workshop to identify and assess ethical issues based on MEESTAR, as well as the intended worth of the system. Next, the worth designer conducts context of use analyses (as in other human-centred design processes), producing Persona stakeholder models and other artefacts. In parallel, the developer engages in a technical exploration phase as in XP in order to find out if, how and with what expected effort specific requirements can be fulfilled and features implemented. Subsequently, the results are consolidated into an initial Worth Map. On this basis the customer and worth designer define superordinate project goals and evaluation metrics, and the customer decides upon a set of features that shall be implemented in the first iteration.

After that, the iterative production phase starts (light-blue ellipse in Fig. 3). The duration of iterations depends on the properties of individual projects and should be kept constant during development. The developer implements a set of features that have previously been defined by the customer and designed and tested by the worth designer. In parallel, the worth designer creates prototypes for a set of features that are supposed to be implemented in the subsequent iteration, and conducts formative evaluations with them. Finally, the three roles get together for a meeting in which the developer reports on the expected costs for implementing pending features, the worth designer updates the Worth Map with relevant new information that has been gathered in the meantime, and the customer then decides upon the features that shall be designed in the next iteration. An important characteristic of AWOSE is that both the selection of features and choices among alternative ways of implementing these should primarily be based on the current Worth Map by evaluating the ethical assessment and expected generation of worth that are associated with each of the still outstanding features. After this meeting, the next iteration starts.

## Discussion

Adequate consideration of ethical aspects in research and development must balance diligence and practical feasibility. As Zhu and Jesiek (2017) noted, “*preferable ethical decisions are “workable”, i.e., they need to be both ethically justifiable and practically plausible*” (Zhu and Jesiek 2017, p. 677). Whenever an agile approach to development can be adapted, the AWOSE methodology may help structure the process and suggest how to apply effective methods to satisfy these requirements.

The two main parts of the methodology, MEESTAR and WCD, complement each other regarding their goals and the insights generated by their application. MEESTAR aims at safeguarding against harm by identifying ethical sensitivities related to a system (Manzeschke et al. 2015). A broad range of projects has demonstrated its applicability, including and beyond age-appropriate assistance systems such as ADAMAAS. The second component of AWOSE, WCD, aims at designing systems that deliver value in the world, which endures after interaction (Cockton 2006), and established the use of worth map diagrams to support this goal during system design. In addition to providing a systematic structure for effective integration of MEESTAR and WCD, AWOSE incorporates several improvements on its heritage. The MEESTAR set of ethical dimensions was extended to include nature-related aspects, such as sustainability of systems, and the important step of determining ethical severity ratings has now been procedurally defined. Worth maps have, as a result of several iterative optimizations, already been described as “state of the art in values focused methods” (Cockton 2012, p. 4). Nonetheless, the fusion with UML diagrams as proposed in this article makes them potentially more useful for complex development projects and may popularize their use in organizations with a strong technocratic orientation. On a final note, using these improved worth maps the AWOSE methodology may also support large-scale system-level analyses as demanded for example by Borenstein et al. (2017) in the context of autonomous driving.

Compared with many other approaches to assessing ethical issues in engineering (e.g. Hofmann et al. 2017; Hofmann 2017; Alkhatib and Abdou 2017; Lokhorst 2018; Kermisch and Depaus 2018), the approach taken in AWOSE has several conceptual benefits. It is comparably “open” in the sense that, albeit referring to a set of high-level ethical dimensions in order to stimulate and structure the brainstorming process, it does not impose a specific pre-defined list of questions that may unduly bias and distort results. Furthermore, it supports the assessment of society-level concerns related to public accountability of research as called for by Matthias Leese (2017), and it is embedded in an overarching process model. On a theoretical level, AWOSE differs from VSD in that it explicitly distinguishes between avoiding ethical issues on the one hand and creating worth on the other hand, according to Herzberg’s two-factor theory. Hereby “not creating worth” (i.e. not increasing stakeholder’s motivation for system use) does not necessarily imply ethical issues, whereas neglecting “hygiene factors” might constitute an ethical issue. Other than VSD’s definition of “values”, AWOSE’s “worth” does not need to be something that people “consider important in life” (Friedman et al. 2008, p. 70), but only something that motivates them to use the system. The VSD approach (Davis and Nathan 2015, p. 22) requires that “designers *must* attend to values supported by theories of right, which are obligatory, and *may* attend to values supported by theories of the good, which are discretionary”. The terminology and methodological approach of AWOSE makes a conceptually related, but more explicit distinction by requiring that (negative) ethical issues *must* be prevented or mitigated, while any kind of (positive) worth *may* be created through a system in order to motivate its usage. For example, “looking hip and stylish” would probably not be considered “important in life” by many people, but nevertheless be a factor motivating them to buy and use such things over less aesthetically pleasing alternatives. In comparison with Spiekermann’s E-SDLC approach, AWOSE embraces agile principles more genuinely. E-SDLC supposes that the prioritization of values is finished before iterative software engineering even starts. In AWOSE’s production phase, worth maps are updated at the end of each iteration. This enables the “customer” to establish priorities for the next iteration with explicit reference to the intended worth and ethical issues, even when the requirements and system architecture have changed arbitrarily since the previous planning meeting. Apart from that, the two methodologies share many conceptual similarities. For example, both approaches refer to Personas as stakeholder models for ethical analyses. Notably, Spiekermann only presents the “Ad-Hoc Persona” variant (as Norman called it), whereas AWOSE prefers data-driven stakeholder models whenever possible. The decomposition or conceptualization of values as described by Spiekermann (i.e. breaking a value “down into the subdimensions that constitute its essence”, p. 205) maps directly to means-end chains of worth elements in AWOSE’s worth maps. Furthermore, AWOSE and E-SDLC (as well as VSD for that matter) concordantly promote choosing design alternatives with respect to their value–worth-related impact, albeit at different points in time within the process.

As of now, practical experience with the AWOSE methodology is limited to research project ADAMAAS. Approaches from AWOSE have been applied in this project to analyze ethical aspects and guide development of the smart glasses assistance system with promising results and to its stakeholders’ satisfaction. However, it remains to be seen how the methodology scales and adapts to larger developments projects and other types of research. From a conceptual and theoretical perspective, AWOSE using MEESTAR’s default dimensions (plus nature-related considerations) certainly suits the development of ICT-based assistance systems best, but it should be applicable to other technical systems and potentially other forms of engineering as well, as long as these allow for rapid prototyping and short iterations during development. In this case, the ethical dimensions and the integration of technical descriptions in worth maps may need to be aligned accordingly. Therefore, further contributions to its appraisal and reports on empiric evidence assessing its suitability in different application contexts are encouraged.
